# Urinary Protein/Creatinine Ratio in Feline Medicine: Reasons to Perform It and Its Role in Clinical Practice—A Retrospective Study

**DOI:** 10.3390/ani12121575

**Published:** 2022-06-18

**Authors:** Maria Ana Fidalgo, Rodolfo Oliveira Leal, José Henrique Duarte-Correia

**Affiliations:** 1 ACIVET/Hospital Escolar Veterinário, Faculty of Veterinary Medicine, University of Lisbon, 1300-477 Lisboa, Portugal; mariaanafidalgo@gmail.com; 2 CIISA—Centre for Interdisciplinary Research in Animal Health, Faculty of Veterinary Medicine, University of Lisbon, 1649-004 Lisboa, Portugal; zeca@fmv.ulisboa.pt; 3 Associate Laboratory for Animal and Veterinary Sciences (AL4AnimalS), 1300-477 Lisbon, Portugal

**Keywords:** proteinuria, UPCR, chronic kidney disease, nephrology, renal biomarkers, urinary tract

## Abstract

**Simple Summary:**

Urinary protein/creatinine ratio allows veterinary clinicians to quantify proteinuria, i.e., the amount of protein that is lost in urine. This ratio is commonly performed in daily practice for several medical reasons, namely the diagnosis and the monitoring of feline chronic kidney disease. This study aimed at understanding the main reasons to perform UPCR in cats, correlating it with signalment, exploring the agreement between UPCR, urine dipstick protein value, and urine specific gravity and assessing its role in chronic kidney disease diagnosis and monitoring. A retrospective study was conducted, including medical data from cats consulted in a veterinary teaching hospital over two years and submitted to UPCR measurement. A total of 140 cats were included: 35% were non-proteinuric, 25% borderline proteinuric, and 40% showed overt proteinuria. This study found that UPCR is mainly requested for the diagnosis and the monitoring of chronic kidney disease and proteinuric cats with kidney disease have a worse outcome at 6-months and at 12-months. This study found and reinforced the negative prognostic value of UPCR in cats in comparison to dipstick and urine specific gravity.

**Abstract:**

This study aimed at understanding the reasons veterinarians conduct a urinary protein/creatinine ratio (UPCR) in cats, correlating it with signalment, dipstick proteinuria tests, and urine specific gravity (USG) and assessing its role in chronic kidney disease (CKD) diagnosis and monitoring. A retrospective study was conducted, including medical data from cats consulted between 2016 and 2018 in a veterinary teaching hospital and submitted to at least one UPCR measurement. A total of 140 cats were included: 35% non-proteinuric (UPCR < 0.2), 25% borderline proteinuric (0.2 < UPCR < 0.4), and 40% overtly proteinuric (UPCR > 0.4). In contrast to other studies, there was no association between UPCR and male reproductive status. UPCR was mainly requested for CKD diagnosis and monitoring. Correlation between UPCR and combined results from dipstick tests and USG was low and inconsistent. Proteinuric CKD cats had a worse outcome at both 6 (odds ratio (OR 4.04) and 12 months (OR 4.36)), and this finding was more pronounced for severely proteinuric cases in which the OR for death was 4.36 and 6.00 at 6 and at 12 months, respectively. In addition to reinforcing the negative prognostic value of proteinuria, this study stresses the low and the inconsistent agreement between UPCR and the combined results of dipstick tests and USG in cats.

## 1. Introduction

Feline proteinuria is defined as the presence of excessive amounts of protein in urine, including albumin (the most prevalent) and globulins [1]. Its clinical assessment relies on the determination of its magnitude, type, origin, and persistence [2,3].

Although there are several diagnostic tests for proteinuria assessment, the International Renal Interest Society (IRIS) recommends using a urine protein/creatinine ratio (UPCR) for CKD substaging. According to this international classification, cats are categorized as non-proteinuric if UPCR < 0.2, borderline proteinuric if 0.2 < UPCR ≤ 0.4, and proteinuric when UPCR > 0.4 [4].

Based on previous studies, proteinuria can be classified as physiological or pathological and, according to its origin, it can be pre-renal, renal, or post-renal [2]. There are some diseases commonly associated with proteinuria in cats, namely chronic kidney disease [5], systemic hypertension [6], hyperthyroidism [7], and lower urinary tract disease [8].

It is thought that, based on previous studies [9,10], intact males had higher levels of proteinuria than those neutered. This is mainly due to the excretion of cauxin, a major protein component highly excreted by intact male cats, which could raise the levels of UPCR [9,10].

Persistent proteinuria has several consequences, and it is a negative prognostic factor in cats with chronic kidney disease [11]. Nowadays, the role of proteinuria in kidney disease is, however, controversial. While some authors believe it is a mere marker of renal lesion, others consider it as a causal agent [12,13,14,15,16]. Although this role is not completely understood, it is generally agreed that veterinary surgeons should value the detection, evaluation, monitoring, and treatment of proteinuric animals [2,3].

The aims of the present study were: to establish the main reasons for UPCR request in feline medicine, to verify if sex or neutered state had any influence on the magnitude of proteinuria, to investigate if there was any correlation between UPCR and creatinine values, to compare results between UPCR and the combined evaluation of dipstick proteinuria tests and urine specific gravity (USG), to stage cats with CKD in the sample according to IRIS criteria and to investigate the clinical outcome at 6 and at 12 months in proteinuric and non-proteinuric animals.

## 2. Materials and Methods

This retrospective study included all cats with at least one UPCR measured in the Clinical Pathology laboratory (Lab Professor Doutor M.Braço Forte), at the Faculty of Veterinary Medicine—University of Lisbon, over three years (between 1 January 2016 and 31 December 2018). All samples collected by cystocentesis were included. UPCR quantification was performed following standard laboratory procedures. After collection and centrifugation, the pyrogallol red molybdate reagent was used for protein measurement and urinary creatinine was assessed by an enzymatic colorimetric assay. Both were measured and creatinine measurements were conducted in an auto-analyzer (Daytona, Randox Laboratories Lda, Lisbon, Portugal), following manufacturer instructions.

All medical records of these cats were reviewed and data was collected, detailing: age, sex, neutered vs. non-neutered, breed, reason for requesting UPCR, magnitude of proteinuria, creatinine, systolic blood pressure, urinalysis namely dipstick proteinuria result (Aution sticks, Menarini, Japan), and USG (determined using a refractometer), and clinical outcome at 6 and at 12 months. Specifically concerning dipstick proteinuria, registered data was obtained following the appropriate semi-quantitative colorimetric scale, from negative (-) to severely proteinuric sample (4+). Among proteinuric cats, urinary sediment was classified as active or inactive according, respectively, to the presence or absence of bacteriuria or >5 leucocytes or erythrocytes per high power-field.

Cats were classified according to IRIS criteria regarding proteinuria in non-proteinuric (if UPCR < 0.2), borderline proteinuric (if UPCR 0.2–0.4), or proteinuric (if UPCR > 0.4).

All statistical analysis was done using IBM SPSS Statistics 20^®^. Data were assessed for normality using the Kolmogorov–Smirnov test. When data was not normally distributed, results were described using median (range). The Mann–Whitney U test was used to compare UPCR values between genders, neutered state, and the association between proteinuria, serum creatinine, and clinical outcome at 6 and at 12 months. *p*-values ≤ 0.05 were considered significant for a confidence interval of 95%. An odds ratio was calculated to determine the risk of death or of euthanasia in proteinuric animals at 6 and at 12 months.

Descriptive statistics took into consideration the number of cats. In cases in which several UPCR were performed over time, only the first time-point value was considered. However, to determine the association between UPCR results and combined dipstick proteinuria and USG results, the total number of urine analyses were used (including those that were performed in the same cat over time). Additionally, to evaluate the main reasons for performing the UPCR in daily practice, the number of UPCR determinations rather than cases was considered.

## 3. Results

### 3.1. Sample Characterization: Proteinuric vs. Non-Proteinuric Cats

In total, 140 cats were included in the study. The median age was 11.5 years old, ranging between seven months and 20 years. There were 72 males (52 neutered) and 68 females (47 neutered). Most cats were domestic shorthair (*n* = 115; 82.14%). Among the pure-breed, 11 Persians, 6 Siamese, 6 Norwegian Forest, 1 Angora, and 1 Ragdoll were identified. There were 49 (35.0%) cases of non-proteinuric animals (median 0.13, range 0.06–0.18), 35 (25.0%) borderline proteinuric cases (median 0.31, range 0.2–0.4), and 56 cases of cats (40.0%) with overt proteinuria (median 0.96, range 0.41–4.17), 17 of which showed severe proteinuria (UPCR > 1). Most proteinuric cats had UPCR values between 0.4 and 1 (*n* = 39; 69.6% of proteinuric cats).

Detailing gender findings: among females, there were 25 (36.8%) non-proteinuric, 16 (23.5%) borderline proteinuric, and 27 (39.7%) overtly proteinuric. The median value of UPC in this group was 0.47 (range 0.06–3.88). Regarding males, there were 24 (33.3%) non-proteinuric, 19 (26.4%) borderline proteinuric, and 29 (40.3%) significantly proteinuric. The median UPCR value was 0.54 (range 0.07–4.17). Males were divided according to their neutered state. Among intact males, there were 8 (40%) non-proteinuric, 5 (25%) borderline proteinuric, and 7 (35%) significantly proteinuric. The median UPCR was 0.51 (range 0.07–2.15). Regarding neutered males, there were 16 (30.8%) non-proteinuric, 14 (26.9%) borderline proteinuric, and 22 (42.3%) significantly proteinuric. The median UPCR was 0.56 (range 0.07–4.17). No statistical differences were found between these groups concerning sex, neutered state in males, and age (males vs. females *p* = 0.89; intact females vs. neutered females *p* = 0.60; intact males vs. neutered males *p* = 0.372, respectively).

### 3.2. Reasons for Performing UPCR

A total of 16 cats in the sample had more than 1 UPCR, totalizing 200 measurements from the 140 cats. Based on the medical records, reasons for requesting this ratio were: diagnosis of CKD (*n* = 105; 52.5%), CKD monitoring (*n* = 41; 20.5%); diagnosis and/or monitoring of concurrent CKD and lower urinary tract disease (*n* = 33; 16.5%), concurrent CKD and hyperthyroidism (*n* = 11; 5.5%), acute kidney disease (AKI) (*n* = 4; 2.0%), CKD and concurrent diabetes mellitus (*n* = 3; 1.50%), and in the context of medical investigation of other systemic diseases (diabetes mellitus, colitis, hypertrophic cardiomyopathy) (*n* = 3; 1.50%).

### 3.3. UPCR vs. Serum Creatinine Concentration

A total of 129 serum creatinine determinations were found. Correlating UPCR with serum creatinine concentration, it was found that proteinuric cats had significantly higher creatinine values when compared to those that were non-proteinuric (including borderline cases) (*p*-value < 0.01). The median value of serum creatinine in non-proteinuric and proteinuric cats was 2.3 mg/dL (range 0.8–9.1) and 3.6 mg/dL (range 1–10), respectively (Table 1).

### 3.4. UPCR vs. Dipstick Proteinuria Results and Urine Specific Gravity (USG)

There were 150 urine analyses available that allowed for a comparison between UPCR values and dipstick results.

Based on urinary dipstick results, there were 84 analyses (56%) that tested negative for proteinuria. From these, when UPCR was concurrently performed, there were 32 (38.1%) non-proteinuric, 16 borderline proteinuric (19.0%), and 36 (42.9%) significantly proteinuric.

**Figure 1:**
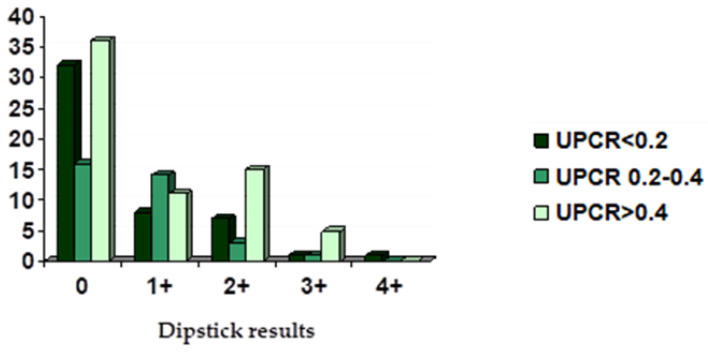
Correlation between UPCR and dipstick results (*n* = 150 urinalyses).

A total of 33 analyses had a 1+ on the dipstick for proteinuria. When UPCR was performed, there were 8 (24.2%) that were non-proteinuric and 14 (42.4%) borderline proteinuric, while 11 (33.3%) were significantly proteinuric. Approximately 25 analyses revealed 2+ on the dipstick proteinuria test. According to late performed UPCR, 7 (28%) were non-proteinuric, 3 (12.0%) borderline proteinuric, and 15 (60.0%) were significantly proteinuric. Concerning 7 analyses that tested 3+ on the dipstick for proteinuria, 1 (14.3%) was negative and 1 was borderline proteinuric (14.3%), while 5 (71.4%) were significantly proteinuric according to UPCR. Results are summarized in Figure 1. Only one cat tested 4+ on the dipstick and it was considered non-proteinuric when a UPCR was performed. However, it is worthwhile to detail that urine from this cat was yellow but its pH was 8.5.

Also, USG was correlated with dipstick and UPC results. Table 2 summarizes the results of both dipstick tests and USG values for the present study and the distribution of proteinuria staging based on UPCR [4].

There were 8 cats with appropriate USG (>1.035) and 2+ to 4+ dipstick results that were non-proteinuric when the UPCR was performed. On the other hand, there were 36 cases with negative dipstick results that were overtly proteinuric according to the UPCR. There were four cats with a low USG (<1.012) and negative dipstick results that were significantly proteinuric.

From the 150 urine analyses, 35 showed an active sediment; among those, only 8 had a positive urine culture (6 proteinuric and 2 non-proteinuric).

### 3.5. UPCR and IRIS Staging

According to the medical records, complete IRIS staging was possible in 61 cases (43.6%). From these, there were 31 proteinuric and 30 non-proteinuric cats (including borderline).

Regarding the non-significant proteinuric group, 2 cats were in stage I (1 was non-hypertensive (non-HT) and 1 was hypertensive (HT)) and 16 cats were in stage II (2 were non-HT and 14 were HT). Eleven cats were in stage III (all were HT) while one cat was in stage IV (with HT).

In the proteinuric group, there were three cats in stage I (1 non-HT, 2 HT), nine cats in stage II (2 non-HT, 7 HT), nine cats in stage III (1 non-HT, 8 HT), and ten cats in stage IV (1 non-HT, 9 HT).

### 3.6. UPCR and Clinical Outcome of CKD Cases at Six and Twelve Months

Clinical outcome at 6 and at 12 months of both non-proteinuric (including borderline) and proteinuric animals is shown in Table 3. There was a significant correlation between UPCR values and clinical outcome at 6 months (*p* = 0.002) and at 12 months (*p* = 0.001). Using the odds ratio, it was possible to observe that in proteinuric animals (UPCR > 0.4), the likelihood of a worse outcome was 4.04× and 4.36× greater than in non-proteinuric animals at 6 and at 12 months, respectively. This was even more evident in severely proteinuric cats, in which the odds ratio values for death or euthanasia at 6 and at 12 months were 4.57× and 6.00×, respectively (Table 4).

## 4. Discussion

This study highlights the main reasons why clinicians requested a UPCR in cats, supporting the diagnosis and the monitoring of CKD as the most frequent.

In this study, cats submitted to UPCR measurement were mainly geriatric, which is in agreement with the fact that most of the diseases that induce proteinuria in cats, namely CKD, are particularly prevalent in elderly ages [5,9,14].

The proportion of proteinuric, non-proteinuric, and borderline-proteinuric cats was relatively homogenous, although proteinuric cats were slightly overrepresented, accounting for approximately 40% of the cases in which UPCR was requested. From these, only a minor percentage was found significantly proteinuric (UPCR > 1), which is in agreement with previous studies supporting that cats tend to present mild to moderate proteinuria [2,17,18], mostly due to tubulointerstitial fibrosis [17,19].

Contrarily to previous publications [9,10], there was no significant correlation between the UPCR values and a neutered state, which questions the role of cauxin on significant proteinuria. Although further studies would be useful to better clarify these findings, in the authors’ opinion, significant proteinuria should be investigated regardless of a neutered state.

In this study, the most relevant reasons to perform UPCR in cats were diagnosis and monitoring of CKD, which accounted for about three quarters of the requested analysis. This is expected, as CKD is highly prevalent among cats and proteinuria forms part of its proper diagnosis and staging [4]. However, when the number of analysis requested for CKD diagnosis are compared with those sought for its monitoring, it was observed that a solid proportion of animals did not have any follow-up analysis. This can be justified by several reasons, detailing: owner’s lack of compliance, loss of follow-up, infrequent use of geriatric panels, or by other unknown reason. Nonetheless, these findings reinforce that clinicians should be sensitized to the relevance of controlling and treating proteinuria [12,13], as it is a marker of progression and a negative prognostic factor in feline CKD cases [5,14,15,16].

Although weak, an association between UPCR and creatinine values (*r* = 0.379) was observed, meaning that increased proteinuria is linked to more severe CKD. This is in line with previous studies supporting the relevance of UPCR in the progression of CKD [6,11,18].

The level of agreement between the results of UPCR and dipstick tests was low. It is interesting to observe that there were both cats with dipstick results of 4+ that were negative for UPCR and there was also a significant number (*n* = 36) of negative dipstick results that were overtly proteinuric according to the UPCR method. Additionally, previous studies [1] state that, when compared to dipsticks, UPCR has a higher specificity (99.2% vs. 11%) and it is not as strongly influenced by other factors (such as USG and urine pH) [5], making it more accurate to diagnose proteinuria. In 2010, Zatelli et al. [20], evaluated the possibility of using the combination of a dipstick test and USG as a replacement for UPCR for identifying proteinuria in dogs. According to their study, negative or 1+ dipsticks and a USG higher than 1.012 dismissed the need to use UPCR to exclude proteinuria. In our study, it was possible to observe that of the 113 cases that should automatically be considered non-proteinuric (according to Zatelli et al., 2010 [20]), only 70 (61.95%) presented UPCR < 0.4, showing that the conclusions from Zatelli’s study would not be applicable for cats. One cat from this study had 4+ on a dipstick and was non-proteinuric according to UPCR. Although the urine was yellow, the urinary pH was 8.5. Since alkaline urine may induce false-positive results on a dipstick proteinuria test, this was considered a false-positive [21,22]. In contrast to dogs, this study highlights that it is not possible to predict levels of feline proteinuria from the combination of dipstick tests and USG. This was also recently suggested by Pérez-Accino et al. [21]. For the reasons stated above, we advise the systematic use of UPCR to diagnose proteinuria in cats, following the International Renal Interest Society recommendation [4,22].

In the present study, IRIS staging was performed in less than half of the cases. Therefore, not all cats in which UPCR was performed in the context of CKD were completely staged and sub-staged. This low proportion reinforces the need for increased awareness for this staging system and, subsequently, a better diagnosis and management of cats with CKD.

Regarding the clinical outcome, a significant correlation between UPCR and survival was found, which agrees with previous studies [17,18,23,24]. Proteinuric cats with CKD have a negative outcome at both 6 and 12 months, which is particularly evident in cats with severe proteinuria. These findings highlight that proteinuria should be closely monitored in all patients with renal disease, being a relevant negative-prognostic factor in a medium–long term perspective.

Although this study has yielded interesting results, it also had a few limitations. Firstly, due to its retrospective nature, the difficulty in standardization of data impaired a better data collection and solid comparisons. Secondly, UPCR assessment was based on one single measurement rather than on a pool of samples, limiting the correct assessment of UPCR variability [25,26,27]. Nonetheless, we believe that serial measurements are routinely unpractical and that they are not always performed in daily practice, meaning that medical decisions are often based on single measurements. Thirdly, there were some data missing, including creatinine concentrations, dipstick tests, systemic blood pressure, UPCR follow-up, and clinical outcome at 6 and at 12 months. We recognize that this can introduce some bias, which has an impact on the power of these results.

It is also worthwhile to mention that the presence of active sediment, although in a minor percentage of cases, may have introduced some bias. Although it is evident that pyuria or macroscopic hematuria can affect proteinuria in dogs [28,29], to the authors’ best knowledge, studies concerning the true effect of active sediment in feline UPCR are lacking. Recognizing that active sediment may potentially increase the true value of UPCR, this effect can be considered a flaw in this study. The fact that cases were collected from a veterinary-teaching hospital could have erroneously led to a referral bias in the selection of cases. However, as primary care service accounts for a large percentage of the caseload from the hospital, the authors believe that these results reflect the reality of feline proteinuria in daily practice.

## 5. Conclusions

Whether a causal agent or a simple marker of CKD, proteinuria has a strong influence on the course of the disease [30,31,32]. In agreement with the previous literature, this study highlights that higher levels of UPCR are associated with more severe stages of CKD.

This study supports that diagnosing and monitoring CKD is the most frequent reason for requesting a UPCR.

No association between proteinuria and a neutered state in males was found, questioning the role of cauxin in intact males, making all increases in UPCR relevant in these animals.

As stated by IRIS, the authors also recommend UPCR as the main tool for diagnosing proteinuria in cats. This study advises against replacing this ratio with the combined evaluation of dipstick tests and USG, as it presented inconsistent results.

Regarding clinical outcome, this study shows that proteinuric cats were approximately 4× more likely to die at 6 and at 12 months, rising up to 6× the hazard ratio in animals with UPCR > 1 in a year. This highlights the importance of closely monitoring and treating proteinuria in cats.

Complete IRIS staging and follow up at 6 and at 12 months was only possible in less than half of the population sample, showing that clinician–owner communication should focus more in linking these variables to progression of their animal’s health.

UPCR measurement is an easy, inexpensive, and a rather sensitive tool for quantifying proteinuria in cats, which allows for the correct diagnosis and monitoring of CKD. More than understanding its role on the progression of CKD, these results reinforce that veterinary surgeons should be aware of proteinuria and explain it to owners in a practical form, including why it is important to monitor this ratio, in order to achieve better quality of life in feline patients.

## Figures and Tables

**Table 1:** Comparison between proteinuric and non-proteinuric cats regarding serum creatinine concentration.

	Non-Proteinuric (UPCR < 0.4)			Proteinuric (UPCR > 0.4)			
	Number of Cats	Median	Range	Number of Cats	Median	Range	*p*-Value
Creatinine (mg/dL)	76	2.31	0.84–9.1	53	3.59	1.00–10	<0.001 *

* Mann–Whitney U test: significant difference—*p* < 0.05.

**Table 2:** Association among dipstick results, USG, and distribution of UPCR in 150 urinalysis.

	Dipstick Results		
USG	0	1+	2+ to 4+
<1.012	0 NP/4 P	0 NP/0 P	0 NP/0 P
1.012–1.034	35 NP/27 P	11 NP/10 P	5 NP/12 P
>1.035	13 NP/5 P	11 NP/1 P	8 NP/8 P

NP—Non proteinuric (including borderline cases) according to UPCR; P—Proteinuric according to UPCR.

**Table 3:** Comparison between proteinuric and non-proteinuric cats regarding clinical outcome at 6 and at 12 months.

	6 Months			12 Months		
	Alive	Lost to Follow Up	Dead	Alive	Lost to Follow Up	Dead
Non-Proteinuric	52 (60.5%)	22 (27.9%)	10 (11.6%)	34 (39.5%)	37 (45.3%)	13 (15.1%)
Proteinuric	18 (33.3%)	24 (40.7%)	14 (25.9%)	12 (22.2%)	24 (40.7%)	20 (37.0%)

**Table 4:** Odds ratio for death or euthanasia in two UPCR cut-offs.

	6 Months	12 Months
	Odds Ratio (CI 95%)	Odds Ratio (CI 95%)
UPCR > 0.4	4.044 (1.53–10.70)	4.36 (1.67–11.38)
UPCR > 1	4.57 (1.29–16.19)	6.00 (1.74–20.65)

## Data Availability

The data presented in this study are available on request from the corresponding author.
